# Twenty-Five Years of Progress—Lessons Learned From JMIR Publications to Address Gender Parity in Digital Health Authorships: Bibliometric Analysis

**DOI:** 10.2196/58950

**Published:** 2024-08-09

**Authors:** Annika Meyer, Thomas Streichert

**Affiliations:** 1 Institute of Clinical Chemistry Faculty of Medicine and University Hospital University Hospital Cologne Cologne Germany

**Keywords:** digital health, medical informatics, authorship, gender distribution, diversity, bibliometric, scientometric, algorithmic bias reduction, gender gap, JMIR Publications, authorships, author, authors, bibliometric analysis, equality, comparison, gender representation, journal, journals, article, articles, Web of Science, control group, comparative analysis, statistical analysis, gender

## Abstract

**Background:**

Digital health research plays a vital role in advancing equitable health care. The diversity of research teams is thereby instrumental in capturing societal challenges, increasing productivity, and reducing bias in algorithms. Despite its importance, the gender distribution within digital health authorship remains largely unexplored.

**Objective:**

This study aimed to investigate the gender distribution among first and last authors in digital health research, thereby identifying predicting factors of female authorship.

**Methods:**

This bibliometric analysis examined the gender distribution across 59,980 publications from 1999 to 2023, spanning 42 digital health journals indexed in the Web of Science. To identify strategies ensuring equality in research, a detailed comparison of gender representation in JMIR journals was conducted within the field, as well as against a matched sample. Two-tailed Welch 2-sample *t* tests, Wilcoxon rank sum tests, and chi-square tests were used to assess differences. In addition, odds ratios were calculated to identify predictors of female authorship.

**Results:**

The analysis revealed that 37% of first authors and 30% of last authors in digital health were female. JMIR journals demonstrated a higher representation, with 49% of first authors and 38% of last authors being female, yielding odds ratios of 1.96 (95% CI 1.90-2.03; *P*<.001) and 1.78 (95% CI 1.71-1.84; *P*<.001), respectively. Since 2008, JMIR journals have consistently featured a greater proportion of female first authors than male counterparts. Other factors that predicted female authorship included having female authors in other relevant positions and gender discordance, given the higher rate of male last authors in the field.

**Conclusions:**

There was an evident shift toward gender parity across publications in digital health, particularly from the publisher JMIR Publications. The specialized focus of its sister journals, equitable editorial policies, and transparency in the review process might contribute to these achievements. Further research is imperative to establish causality, enabling the replication of these successful strategies across other scientific fields to bridge the gender gap in digital health effectively.

## Introduction

On International Women’s Day 2024, the World Health Organization highlighted the promotion of “gender equality through digital health” [1]. In industrialized countries, women are the leading users of digital health apps [2], wearable devices [3], and their combined applications [4]. Fulfilling the role of “digital carer,” they use these tools not only for their own personal health management but also to attend to the health needs of family members under their care [5]. Despite their predominant role as the principal consumers of digital health resources, a mere 3% of the 2728 digital health deals in the United States from 2011 to 2020 were specifically designed with women in mind [6]. One notable manifestation of this discrepancy is seen in the performance of activity trackers, which are most accurate when worn close to the body. However, the common absence of pockets in women’s clothing often necessitates storing these devices in handbags, thereby potentially compromising their accuracy [7].

Within this framework, research plays an essential role in driving forward digital health innovations and promoting gender equality [8,9]. It is a fundamental component of the approach to “setting norms, developing evidence-based technical guidance and formulating direction to support decision-making in digital health,” which is the strategic priority 1 of the Regional Digital Health Action Plan for the World Health Organization European Region 2023-2030 [10]. Consequently, the Organisation for Economic Co-operation and Development advocates for diversity in research teams as a means of closing the digital gender divide [11]. Creating such teams is vital for understanding societal challenges [7,9,12], enhancing performance [11], and mitigating bias in algorithms [7,11], prompting calls for more detailed investigations into diversity within digital health academia [7].

In response to this identified need in the literature, this study’s objective is to provide a detailed investigation of gender distribution among authors in digital health, with a principal focus on the publisher JMIR Publications. JMIR Publications originated from the *Journal of Medical Internet Research*, an open access pioneer that published its first issue in 1999 [13]. Since then, JMIR Publications has become a leading publisher in the field of digital health, annually releasing more than 3500 papers distributed among more than 30 journals [14].

By examining the practices of JMIR Publications as a positive example, this research intends to identify factors predicting female authorship in the digital health academic community.

## Methods

### Inclusion Criteria

The selection criteria for our study included digital health journals listed in Web of Science and papers published between the beginning of 1999 and end of 2023. For the primary group of interest, we examined all journals indexed by JMIR Publications [15], using their ISSN numbers to search the Web of Science Master Journal List as of February 20, 2024. For any journals not listed in Web of Science, we conducted validation checks on the journals’ official websites.

**Figure 1:**
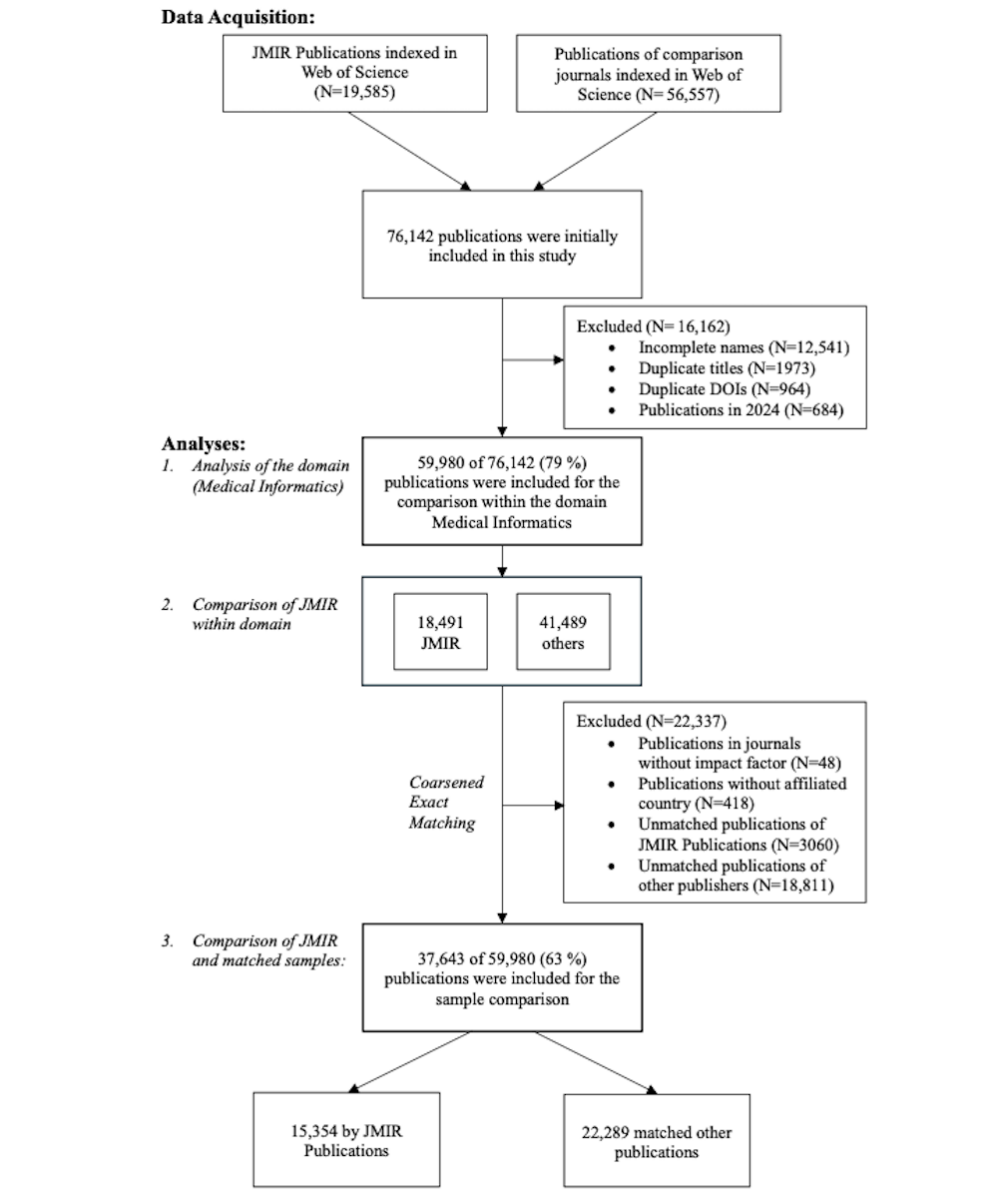
Inclusion and exclusion criteria for publications in the field of medical informatics, 1999-2023.

To maintain consistency across the groups in our study, we selected journals for the control group by applying the filter “Medical Informatics” in the Journal Citation Report. This criterion was chosen because JMIR Publications’ flagship journal, *Journal of Medical Internet Research*, is classified under the same category in the Journal Citation Reports. By using this approach, we were able to identify 15 journals from JMIR Publications and 27 control journals. Following the extraction of metrics such as impact factor and quartile ranking from the Journal Citation Report (Multimedia Appendix 1), we filtered by ISSN and eISSN to identify a total of 76,142 publications in the Web of Science database on February 20, 2024. Following the automated removal of duplicates, incomplete names, and publications after 2023 using the R programming language (R Core Team, 2023) [16], 59,980 publications (79% of the initial data set) were retained for final analysis (Figure 1).

### Gender Assignment

First and last authors’ gender was determined based on their first names using the R programming language [16]. These authorship positions were specifically chosen due to their visibility and relevance within scholarly publishing [17,18]. To ensure accuracy, names with apostrophes were verified against the corresponding publications, resulting in corrections for 10 names. When a secondary name was provided in brackets, the bracketed name was preferred to reduce assignment errors. An automated mixed methods approach was adopted to categorize authorship into female, male, and unknown, combining genderize.io [19] and the Wiki-Gendersort name list [20], as recommended by Sebo [21]. Although this approach oversimplifies the concept of gender and does not fully capture diversity, it was a necessary compromise to facilitate the analysis of trends in authorship distribution.

### Analysis Process

Our analysis then proceeded in 3 stages: first, we evaluated the gender distribution of first and last authors within digital health research. Next, we compared gender proportions between JMIR Publications and others in the field. Finally, we conducted a comparative analysis of JMIR Publications against a control sample of publications in digital health (Figure 1). As is recommended for scientometric studies [22], the sample of 37,643 publications, representing 63% of the initial 59,980, was selected using coarsened exact matching.

This approach aimed to ensure comparability while minimizing the impact of confounding variables, such as publication year, impact factor, document type, international collaboration, and affiliated country. Results of an alternative matching strategy using nearest neighbor matching for impact factors and exact matching for publication year, document type, international collaboration, and affiliated country can be found in Multimedia Appendix 2.

### Statistical Analysis

Continuous variables (publication year, impact factor, usage count, and total times cited) were summarized using means and SDs, whereas categorical variables (quartile ranking, international collaboration, document type, and authors’ gender) were described by frequencies and percentages.

Acknowledging the limitations of normal distribution testing and the robustness of parametric tests in large samples [23], we assumed normal distribution in accordance with the central limit theorem [24]. Differences in continuous variables were analyzed using 2-tailed Welch 2-sample *t* tests, while the Wilcoxon rank sum test was used for ordered categorical variables and chi-square tests for nominal variables. The analysis was further enhanced by calculating odds ratios (ORs) via logistic regression. We defined “Author Concordance” as a variable indicating gender alignment between the first and last authors (both female, both male, or both unknown). In addition, “female in contrasting author position” was categorized as a binary variable, with a value of “true” assigned if both the first and last authors were female and a value of “false” otherwise. To account for the large sample size, the α value for statistical significance was set at <.005, following the recommendations by Di Leo and Sardanelli [25].

We conducted our statistical analysis using the R programming language [16] and using the “rio” [26], “here” [27], “bibliometrix” [28], and “parallel” [16] packages for data import and processing. The “tidyverse” [29] and “fastDummies” [30] packages supported data manipulation, and the “MatchIt” [31] package enabled coarsened exact matching. Tables were generated using the “gtsummary” [32], “knitr” [33], and “labelled” packages [34], and “dplyr” (included in “tidyverse”) as well as “cowplot” [35] was used for graphical presentation.

### Ethical Considerations

All aspects of this research project comply with the ethical standards of the Ethics Committee of the University Hospital Cologne, which reviewed and approved the project on July 2, 2023 (application ID: 22-1436-retro).

## Results

### Publication Characteristics

Our analysis included 59,980 publications, with 18,491 from JMIR Publications and 41,489 from control journals in the field of medical informatics. The average publication year for JMIR papers was notably recent 2020 (3) compared with 2017 (5) for other publications in the field (*P*<.001). JMIR Publications also differed from others in medical informatics in terms of international collaborations (27% vs 25%, respectively, *P*<.001) as well as impact factor for 2022 (5 [2] vs 4 [3], respectively, *P*<.001). Table 1 provides additional distinctions, including usage frequency and citation metrics.

**Table 1:** Summary table of publications published in the field of medical informatics between 1999 and 2023^a^.

Characteristic			Overall (59,980/59,980, 100%)^b^		JMIR, (18,491/59,980, 31%)^b^		Others in medical informatics (41,489/59,980, 69%)^b^		*P* value	
Publication year (SD)			2018 (4)		2020 (3)		2017 (5)		<.001^c^	
**Quartile ranking, n/n (%)**										<.001^d^
	No quartile	6057/59,980 (10)		6057/18,491 (33)		0/41,489 (0)				
	Quartile 1	19,382/59,980 (32)		8001/18,491 (43)		11,381/41,489 (27)				
	Quartile 2	11,910/59,980 (20)		3424/18,491 (19)		8486/41,489 (20)				
	Quartile 3	10,126/59,980 (17)		1009/18,491 (5.5)		9117/41,489 (22)				
	Quartile 4	12,505/59,980 (21)		0/18,491 (0)		12,505/41,489 (30)				
Impact factor 2022, mean (SD)			4 (3)		5 (2)		4 (3)		<.001^e^	
Usage count (since 2013), mean (SD)			14 (24)		15 (26)		14 (23)		.20^c^	
Total times cited, mean (SD)			20 (70)		18 (49)		21 (77)		<.001^c^	
**International collaboration, n (%)**										<.001^d^
	No	44,486/59,980 (74)		13,485/18,491 (73)		31,001/41,489 (75)				
	Yes	15,494/59,980 (26)		5,006/18,491 (27)		10,488/41,489 (25)				
**Document type, n (%)**										<.001^d^
	Paper	52,685/59,980 (88)		15,819/18,491 (86)		36,866/41,489 (89)				
	Editorial	1604/59,980 (2.7)		101/18,491 (0.5)		1503/41,489 (3.6)				
	Letter	549/59,980 (0.9)		141/18,491 (0.8)		408/41,489 (1.0)				
	Other	674/59,980 (1.1)		316/18,491 (1.7)		358/41,489 (0.9)				
	Review	4468/59,980 (7.4)		2114/18,491 (11)		2354/41,489 (5.7)				

^a^Statistical significance is highlighted by *P* values in boldface.

^b^Mean (SD) or frequency (%).

^c^Two-tailed Welch 2-sample *t* test.

^d^Pearson chi-square test.

^e^Wilcoxon rank sum test.

### Gender Distribution of Female Authorship

**Figure 2:**
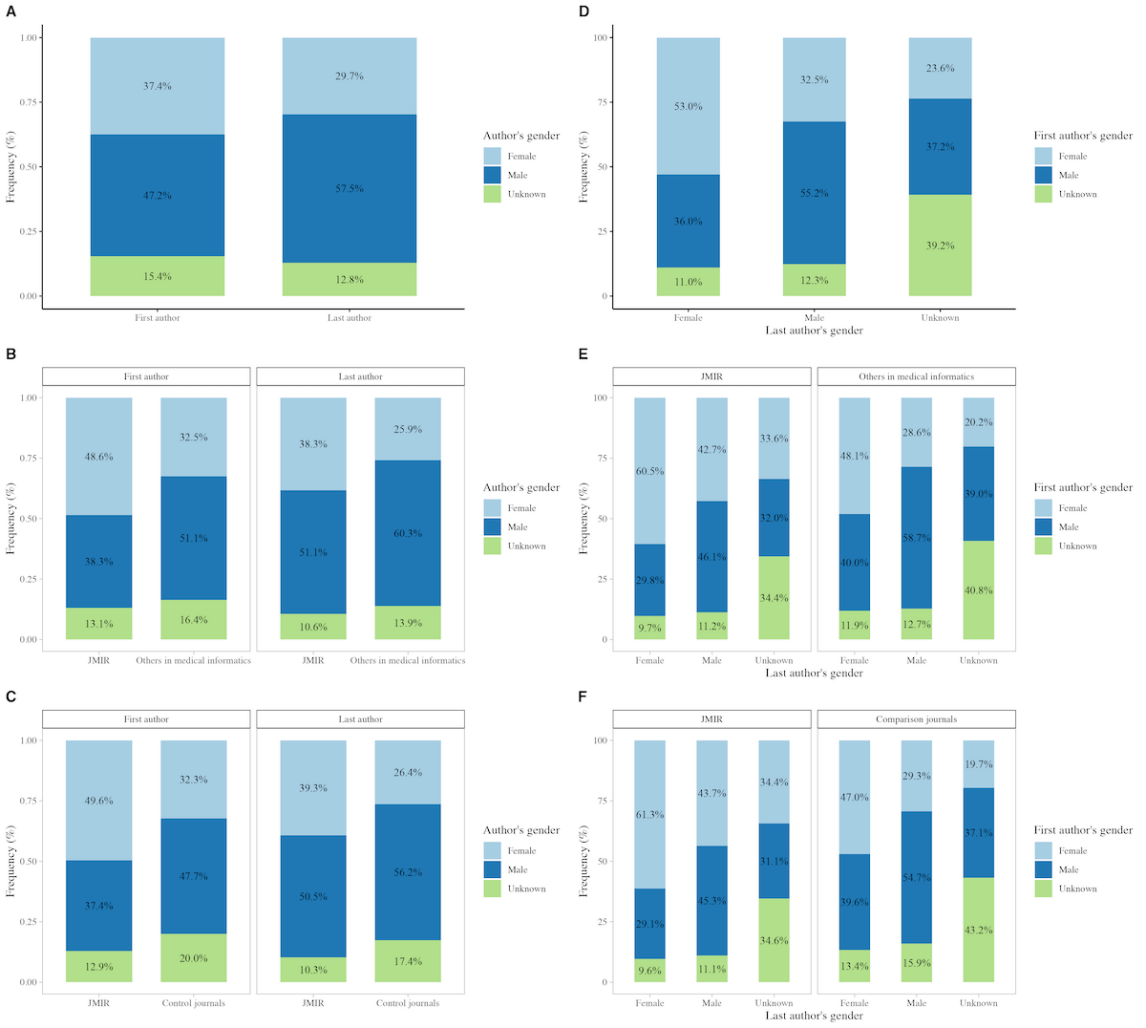
Comparison of gender distribution and gender concordance in first and last authorship within medical informatics in regard to JMIR Publications, 1999-2023. The bar charts display the gender distribution as well as gender concordance in medical informatics (gender distribution: 2A; gender concordance: 2D), JMIR Publications within this field (gender distribution: 2B; gender concordance: 2E), and JMIR Publications compared with the sample of control publications after applying the sampling strategy (gender distribution: 2C; gender concordance: 2F). The proportion of male authors is shown in dark blue, female authors in light blue, and unknown authors in light green.

Among the analyzed publications in medical informatics, the gender distribution of first authors was 37% (22,450/59,980) female and 47% (28,299/59,980) male, while 30% (17,811/59,980) of last authors were female and 57% (34,463/59,980) male. Unspecified gender accounted for the remaining 13%-15% in authorship positions. In contrast to others in medical informatics, publications from JMIR Publications showed a significantly higher proportion of female authors in relevant positions, with 49% (8980/18,491) of first authors (*P*<.001) and 38% (7078/18,491) of last authors being female (*P*<.001). This statistically significant difference was further validated after applying the sampling strategy (first authorship: *P*<.001; last authorship: *P*<.001) (Multimedia Appendix 3; Figures 2A-2C).

### Predictors of Female Authorship

The strongest predictor of female authorship was the presence of female authors in contrasting authorship positions (OR 2.53, 95% CI 2.44-2.62; *P*<.001; Table 2).

Specifically, the proportion of female first authors was 53% (9444/17,811) when the last author was female, compared with only 32% (11,188/34,463) when the last author was male (*P*<.001; Figures 2D-2F). However, as the majority of last authors (34,463/59,980, 57%) were male, gender concordance emerged as a negative predictor of female first authorship (OR 0.51, 95% CI 0.49-0.53; *P*<.001).

Notably, the publisher JMIR Publications served as the second most significant predictor of both female first (OR 1.96, 95% CI 1.90-2.03; *P*<.001) and last authorship (OR 1.78, 95% CI 1.71-1.84; *P*<.001; Table 2). Even after applying the matching strategy, this association remained significant (Multimedia Appendix 4).

**Figure 3:**
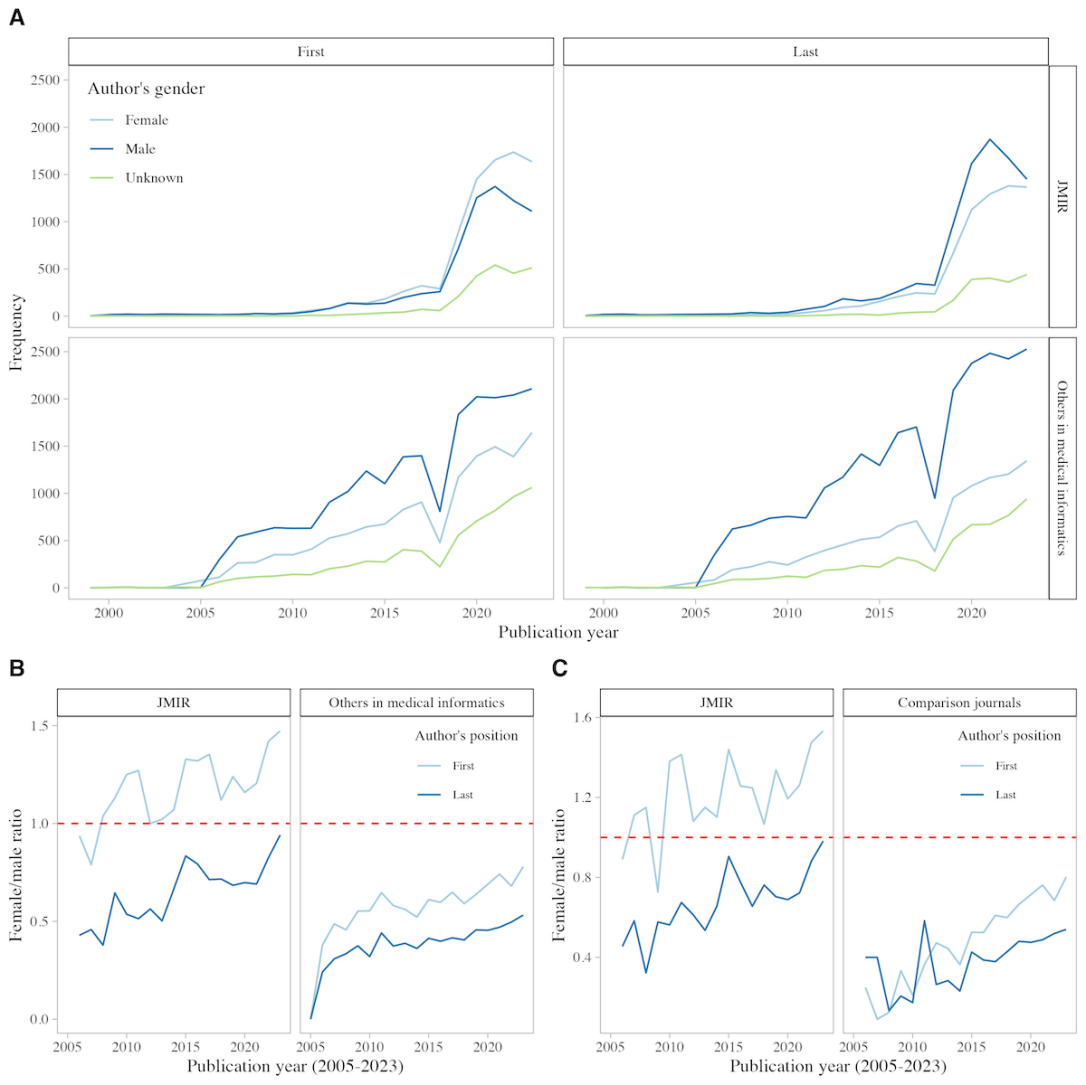
Gender distribution of authorship in medical informatics over time, 1999-2023. The line graph in Figure 3A shows the proportion of female (light blue), male (dark blue), and unknown (green) authors of JMIR Publications and other publications in the field, as well as first and last authors. Line graphs in Figure 3B and Figure 3C show the proportion of male and female first authors (light blue) and last authors (dark blue) for the comparison of JMIR Publications in the field and in the control group. The red line shows gender parity with a ratio of 1:1. JMIR: Journal of Medical Internet Research.

This trend is also reflected over time, with the ratio of female-to-male authors in JMIR Publications exceeding 1:1 since 2008 (Figure 3).

**Figure 4:**
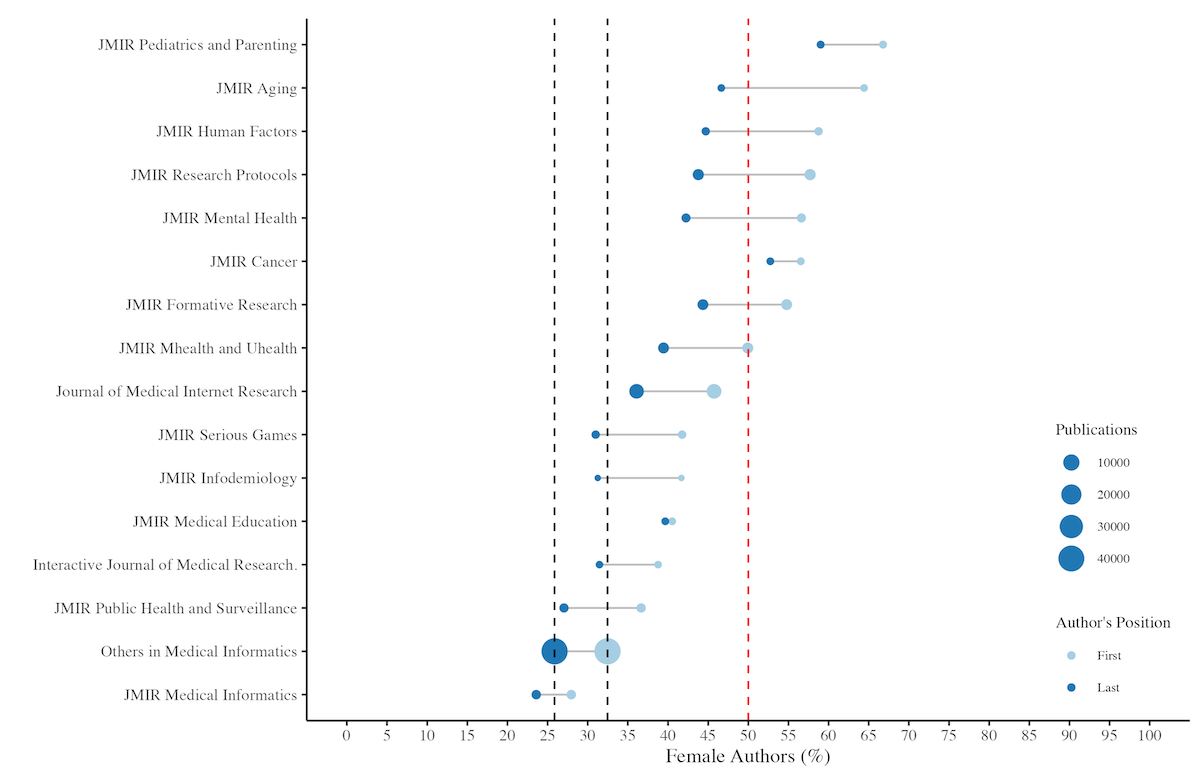
Gender distribution of authorships in medical informatics by journal, 1999-2023. This lollipop chart illustrates the percentage of female authors in first (light blue) and last author positions (dark blue) across various medical informatics journals. The red line signifies the 50% mark, indicating gender parity. The size of the lollipops correlates with the number of publications, providing a visual comparison of female authorship prevalence in the field. The black dashed line on the right indicates the proportion of female last authors, while the line on the left represents the proportion of female first authors in other medical informatics publications. JMIR: Journal of Medical Internet Research.

In addition, 7 JMIR Publications journals had more than 50% female first authors and only 1 had fewer female authors in relevant positions than the pooled analysis of control journals in the field (Figure 4).

**Table 2:** Odds ratios for female first and last authorship in the field of medical informatics between 1999 and 2023^a^.

Characteristic (N=59,980)	First female authorship (N=22,450)				Last female authorship (N=17,811)		
	OR^b^	95% CI	*P* value	OR		95% CI	*P* value
Female in contrasting author position	2.53	2.44-2.62	*<.001*	2.53		2.44-2.62	*<.001*
Gender concordance	0.51	0.49-0.53	*<.001*	1.03		1.0-1.07	.10
Publication year	1.03	1.03-1.04	*<.001*	1.04		1.03-1.04	*<.001*
*Journal of Medical Internet Research*	1.96	1.90-2.03	*<.001*	1.78		1.71-1.84	*<.001*
Impact factor 2022	0.96	0.96-0.97	*<.001*	0.96		0.96-0.97	*<.001*
International collaboration	0.88	0.85-0.92	*<.001*	0.81		0.77-0.84	*<.001*

^a^Statistical significance is highlighted by *P* values in italics.

^b^OR: odds ratio.

## Discussion

### Current Status of Female Authorship in Digital Health

The potential of diverse research teams to mitigate the digital health divide [7,9,11] and as such promote gender equality is increasingly recognized [1].

While the proportion of female authors in medical informatics remains below the 41% observed in the broader field of medicine, there has been a notable increase since 2018. At that time, women accounted for only 33% of first authors and 27% of last authors in this domain [36]. The low, albeit increasing, proportion of female authorship is also reflected at symposia [12,37,38] and within the submission as well as reviewer distribution of leading digital health journals [39]. This gender distribution might suggest biases against women researchers, highlighting the broader structural issues within the digital health sector, such as underrepresentation in leadership roles [7,40] and their disproportionate workload relative to recognition [7]. Thus, professional isolation, including exclusion from networks [40] and the decision-making processes [7,40], may play a more influential role than the often-assumed work-family balance [40]. Consistent with the literature [36], the observed low rate of international collaborations among female scientists and the higher frequency of female authorship in scenarios where women occupy the opposite author position seem to support this hypothesis.

While gender parity remains an aspiration yet to be fully realized in digital health, the current upward trend in female authorship deserves emphasis [12,36-38]. Factors contributing to this positive development include the increasing representation of women in the wider scientific community [36,41] and the implementation of targeted incentives and support initiatives, such as those offered by the American Medical Informatics Association [37,42].

### Innovative Practices and Their Impact on Gender Representation

However, attributing the higher representation of female authors in relevant authorship positions solely to these factors cannot fully account for this trend. JMIR Publications achieving gender parity in first authorship 15 years ago, a milestone predicted to take 27 years to achieve [36], may provide insights into potential strategies to promote female representation in digital health research.

First, the publisher has established niches within various digital health topics through its sister journals [43], offering a specialized forum where competition is confined to works of similar thematic content. Considering the broad and multifaceted nature of the digital health field [44], this approach avoids the challenge of reflecting diversity in research topics and teams within a single journal.

Second, the origin of JMIR Publications as a “small independent open access project hosted at a university” [14] may have facilitated more diverse authorship without the need to conform to traditional and potentially biased structures. For instance, a study from 2021 shows that female editors are underrepresented in 86 medical journals with an average rate of 27.9% [45]. In contrast, JMIR Publications’ flagship journal, *Journal of Medical Internet Research*, currently has a higher proportion of female authors at 35% [46]. At *JMIR Pediatrics and Parenting*, the proportion of female editors is as high as 40%. Among their former editors 80% are female [46]. Given the likelihood of gender concordance between editors and authors in other disciplines [47], lower female presence in editorial boards may also hinder female representation in prominent digital health authorship positions. Reasons for this may include fewer invitations to submit for female researchers [36], unconscious bias in paper evaluation [47], and male editors’ preference for male reviewers [48].

Third, JMIR Publications review process might also mitigate the reviewer bias female authors face in science [49-51]. This bias is often facilitated by the one-sided anonymous nature of single-blinded peer review, which remains the most common form of this process [52]. JMIR Publications, however, uses a signed peer review process [53], which acknowledges reviewers’ work by publishing their names after paper publication [54,55]. As a potential side effect, disclosing the identity of reviewers can also enhance the quality and civility of peer review [56], potentially mitigating bias and thus promoting greater representation of female authors in relevant authorship positions.

Fourth, JMIR Publications offers an expedited review process for a fee [55]. This possibility might be interesting, especially considering that 38% of women in computer science, a field closely related to digital health, report experiencing slower career progression than their colleagues who completed their final degrees at the same time [57]. Female-authored papers tend to spend an additional 3-6 months in the review pipeline compared with male-authored papers, even when they score higher on readability [58]. As merit is often associated with being the first to publish [59], the availability of a faster review process might attract female authors to JMIR Publications. However, it is crucial to acknowledge that while expedited review offers benefits, it must not compromise the integrity of the peer review process, which serves as a critical instrument for quality assurance in academic publishing.

While the gender distribution of JMIR Publications is encouraging and the identified strategies might seem promising, further research to establish causality is essential in order to validate and subsequently apply the successful practices of JMIR Publications to other areas of science.

### Conclusions

The landscape of digital health is witnessing an encouraging shift toward gender parity, with JMIR Publications playing a pivotal role in amplifying the visibility of female authors in relevant authorship positions. This notable achievement may stem from the creation of specialized niches within JMIR Publications’ array of sister journals as well as its publishing practices, such as the transparent disclosure of reviewer identities. Future research into the context of JMIR Publications’ high proportion of female authors is therefore needed to promote diversity in research teams so that research can act as a bridge in the digital health divide.

### Limitations

The study design has some limitations. Due to the retrospective nature of the data collection, no conclusions can be drawn about causality. In addition, the data are not necessarily representative of other scientific fields or publications in other databases such as Scopus or PubMed. The binary approach to gender assignment is also not representative of actual gender diversity and neglects biases due to other marginalized group assignments such as ethnicity. The accuracy of the gender assignment approach depends on the geographical origin of the names, which may hypothetically lead to bias. It is also likely that not all confounding factors have been identified and corrected for.

## Appendix

Multimedia Appendix 1 List of included journals in the field of Medical Informatics.

Multimedia Appendix 2 Comparison of JMIR Publications to Others in Medical Informatics after applying nearest neighbour and exact sampling strategy.

Multimedia Appendix 3 Gender Distribution of Authorship within Medical Informatics as well as after applying the sampling strategy.

Multimedia Appendix 4 Odds Ratios of Female First and Last Authorship after Applying the Exact Sampling Strategy.

## Abbreviations

OR: odds ratio
