# Supplementary material for: Twenty-Five Years of Progress—Lessons Learned From JMIR Publications to Address Gender Parity in Digital Health Authorships: Bibliometric Analysis
Source: J Med Internet Res. 2024 Aug 9;26:e58950. doi: 10.2196/58950 (PMC11344179; doi:10.2196/58950)

Appendix: Exact Matching

2024-03-07

Methods

Libraries

The statistical analysis was coded using the R programming language. The packages “tidyverse” and “fastDummies” were used for data processing, while “MatchIt” enabled the coarsened exact matching for the sampling strategy. Tables were created with the packages “gtsummary” and “labelled”, while the packages “dplyr” (included in “tidyverse”) and “cowplot” were used for the graphical representation.

Data Preparation

1. Importing the cleaned data set

A pre-processed data set was imported in which inclusion and exclusion criteria had already been applied. For data protection reasons, the names of authors were also previously removed from the data set. Moreover, names of journals not published in JMIR were renamed “Control Journals”.

2. Preparing matched data set

To additionally analyse publications of JMIR Publications in relation to comparable publications in Medical Informatics, coarsened exact matching was performed according to the recommendations for scientiometric research (Bittmann, 2021). The aim was to establish comparability while minimising the confounding variables Publication Year, Impact Factor, Document Type, International Collaboration and affiliated country.

The matching strategy had the following characteristics:

```
## A matchit object
##  - method: 1:1 nearest neighbor matching without replacement
##  - distance: Propensity score
##              - estimated with logistic regression
##  - number of obs.: 59514 (original), 31970 (matched)
##  - target estimand: ATT
##  - covariates: PY, DT, JIF, international_collaboration, AU1_CO
```

Further summary of balance can be found in the appendix.

Statistics

1. Summary Statistics

Mean and standard deviation were used to represent continuous variables, while frequencies and percentages were used for categorical variables. Due to the susceptibility of statistical tests to errors and the robustness of parametric tests with large sample sizes (Ghasemi A, 2012), we assumed normal distribution in accordance with the central limit theorem (Kwak, 2015). Therefore, we used t-tests to analyse differences in continuous variables. In contrast, we used the Mann-Whitney U test to examine differences in ordered categorical variables, while we employed chi-squared tests for nominal variables due to the large sample size.

2. Odds Ratio

The analysis was then strengthened by the inclusion of odds ratios. To account for the large sample size, statistical significance was assumed at p < 0.005, following Di Leo’s recommendations (Di Leo, 2020).

Plotting Data

Data is plotted as follows: + Bar Diagram for authorship distribution

- Authorship distribution in journals
- Line graph of authorship over time
- Line diagram displaying authorship distribution over time

Results

1. Publication Characteristics

Our analysis included 60,664 publications, with 18,491 from JMIR Publications and 41,489 from other journals in the field of Medical Informatics. The average publication year for JMIR articles was notably recent 2,020 ± 3 compared to 2,017 ± 5 for the others (p<0.001). JMIR Publications also differed from its competitors in terms of international collaborations (5,006 / 18,491 (27%) vs. 10,488 / 41,489 (25%), p<0.001) as well as impact factor for 2022 (5 ± 2 vs. 4 ± 3, p<0.001). Table 1 provides additional distinctions, including usage frequency and citation metrics.

Publication Characteristics Domain Comparison

Characteristic	Overall, N = 59,980[1]	JMIR, N = 18,491[1]	Others in Medical Informatics, N = 41,489[1]	p-value
Publication Year	2,018 ± 4	2,020 ± 3	2,017 ± 5	**<0.001**[2]
Quartile Ranking				**<0.001**[3]
No Quartile	6,057 / 59,980 (10%)	6,057 / 18,491 (33%)	0 / 41,489 (0%)	
Quartile 1	19,382 / 59,980 (32%)	8,001 / 18,491 (43%)	11,381 / 41,489 (27%)	
Quartile 2	11,910 / 59,980 (20%)	3,424 / 18,491 (19%)	8,486 / 41,489 (20%)	
Quartile 3	10,126 / 59,980 (17%)	1,009 / 18,491 (5.5%)	9,117 / 41,489 (22%)	
Quartile 4	12,505 / 59,980 (21%)	0 / 18,491 (0%)	12,505 / 41,489 (30%)	
Impact Factor 2022	4 ± 3	5 ± 2	4 ± 3	**<0.001**[4]
Usage Count (Since 2013)	14 ± 24	15 ± 26	14 ± 23	0.195[2]
Total Times Cited Count	20 ± 70	18 ± 49	21 ± 77	**<0.001**[2]
International Collaboration				**<0.001**[3]
FALSE	44,486 / 59,980 (74%)	13,485 / 18,491 (73%)	31,001 / 41,489 (75%)	
TRUE	15,494 / 59,980 (26%)	5,006 / 18,491 (27%)	10,488 / 41,489 (25%)	
Document Type				**<0.001**[3]
Article	52,685 / 59,980 (88%)	15,819 / 18,491 (86%)	36,866 / 41,489 (89%)	
Editorial	1,604 / 59,980 (2.7%)	101 / 18,491 (0.5%)	1,503 / 41,489 (3.6%)	
Letter	549 / 59,980 (0.9%)	141 / 18,491 (0.8%)	408 / 41,489 (1.0%)	
Other	674 / 59,980 (1.1%)	316 / 18,491 (1.7%)	358 / 41,489 (0.9%)	
Review	4,468 / 59,980 (7.4%)	2,114 / 18,491 (11%)	2,354 / 41,489 (5.7%)	

[1] Mean and std.-deviation or frequency (%)
[2] Welch Two Sample t-test
[3] Pearson’s Chi-squared test
[4] Wilcoxon rank sum test

2. Gender Distribution in Authorship

Among the analyzed publications in Medical Informatics, the gender distribution of first authors was 22,450 / 59,980 (37%) female and 28,299 / 59,980 (47%) male, while 17,811 / 59,980 (30%) of last authors were female and 34,463 / 59,980 (57%) male. Unspecified gender accounted for the remaining 9,231 / 59,980 (15%)-7,706 / 59,980 (13%) in authorship positions. In contrast, publications from JMIR Publications demonstrated a significantly higher proportion of females in relevant authorship positions, with 8,980 / 18,491 (49%) of first author (p<0.001) and 7,078 / 18,491 (38%) of last authors being female (p<0.001). This statistically significant difference was further validated after applying the matched sampling strategy (first authorship: p<0.001; last authorship: p<0.001) (Appendix_Author_Summary, Figure 2A, Figure 2C, Figure 2D).

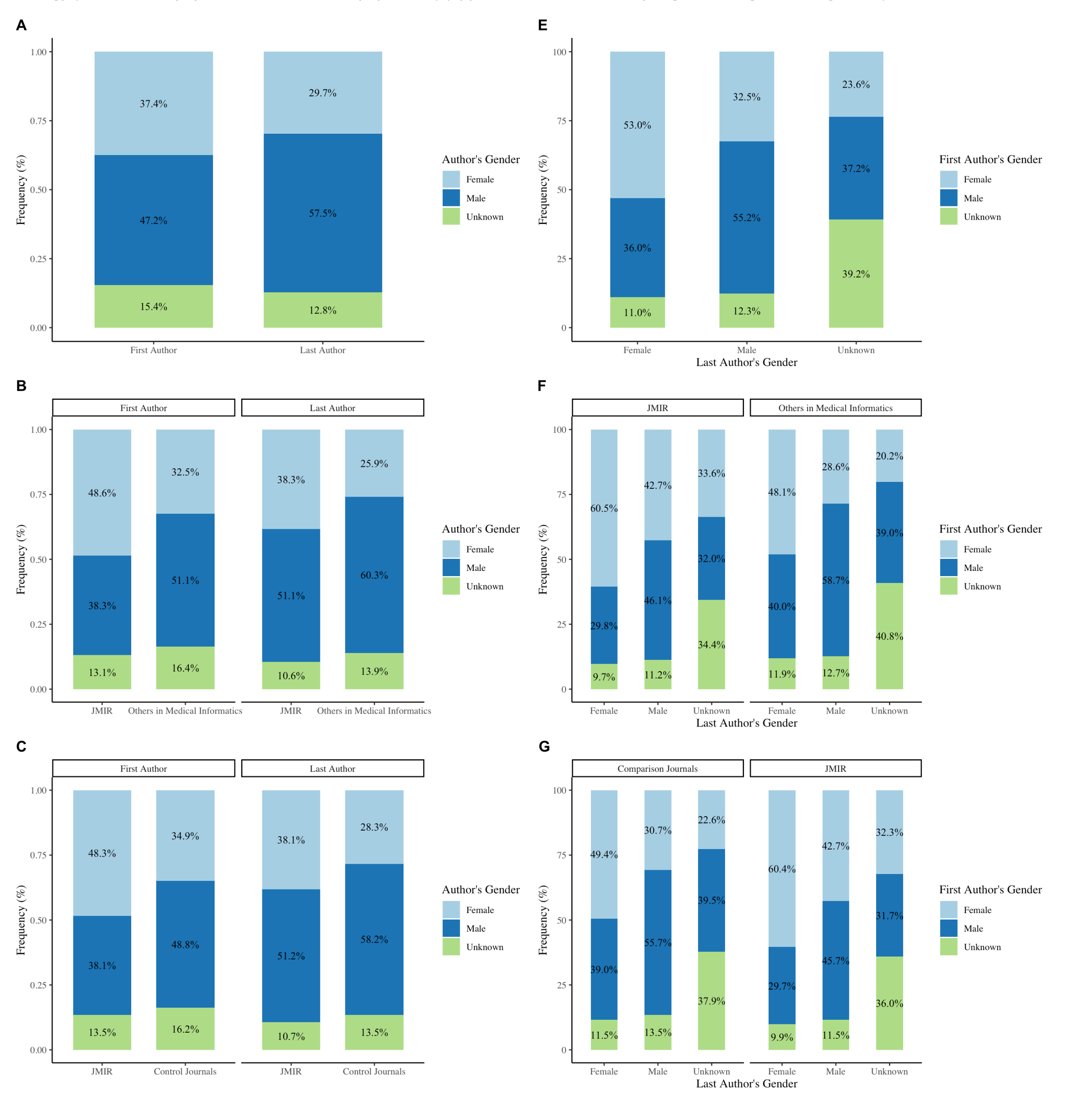

3. Predictors of Female Authorship

The strongest predictor for female authorship was the presence of female authors in contrasting authorship positions (OR: 2.53 (95% CI 2.44, 2.62; p<0.001)) (Table 2).

Odds Ratio for female authorship in Medical Informatics

	First Female Authorship					Last Female Authorship				
**Characteristic**	**N**	**Event N**	**OR**[1]	**95% CI**[1]	**p-value**	**N**	**Event N**	**OR**[1]	**95% CI**[1]	**p-value**
**Female in Contrasting Author Position**	59,980	22450	2.53	2.44, 2.62	**<0.001**					
**Gender Concordance**	59,980	22450	0.51	0.49, 0.53	**<0.001**	59,980	17811	1.03	1.0, 1.07	0.10
**Publication Year**	59,980	22450	1.03	1.03, 1.04	**<0.001**	59,980	17811	1.04	1.03, 1.04	**<0.001**
**JMIR**	59,980	22450	1.96	1.90, 2.03	**<0.001**	59,980	17811	1.78	1.71, 1.84	**<0.001**
**Impact Factor 2022**	59,932	22430	0.96	0.96, 0.97	**<0.001**	59,932	17796	0.96	0.96, 0.97	**<0.001**
**International Collaboration**	59,980	22450			**<0.001**	59,980	17811			**<0.001**
FALSE			—	—				—	—	
TRUE			0.88	0.85, 0.92				0.81	0.77, 0.84	
**Female in Contrasting Author Position**						59,980	17811	2.53	2.44, 2.62	**<0.001**

[1] OR = Odds Ratio, CI = Confidence Interval

Specifically, the proportion of female first authors was 9,444 (53%) when the last author was female, compared to only 6,405 (36%) when the last author was male (p<0.001) (Figure 2B, Figure 2D, Figure 2F). Thus, gender concordance emerged as a negative predictor of female first authorship (OR: 0.51 (95% CI 0.49, 0.53; p<0.001)). Notably, published by JMIR Publications served as the second most significant positive predictor of both female first (OR: 1.96 (95% CI 1.90, 2.03; p<0.001)) and last authorship (OR: 1.78 (95% CI 1.71, 1.84; p<0.001)) (Table 2). Even after exact matching, this association remained significant (Appendix_regression_author_matched). This trend is also reflected over time, with the ratio of female to male authors in JMIR publications exceeding 1:1 since 2008 (Figure 3).

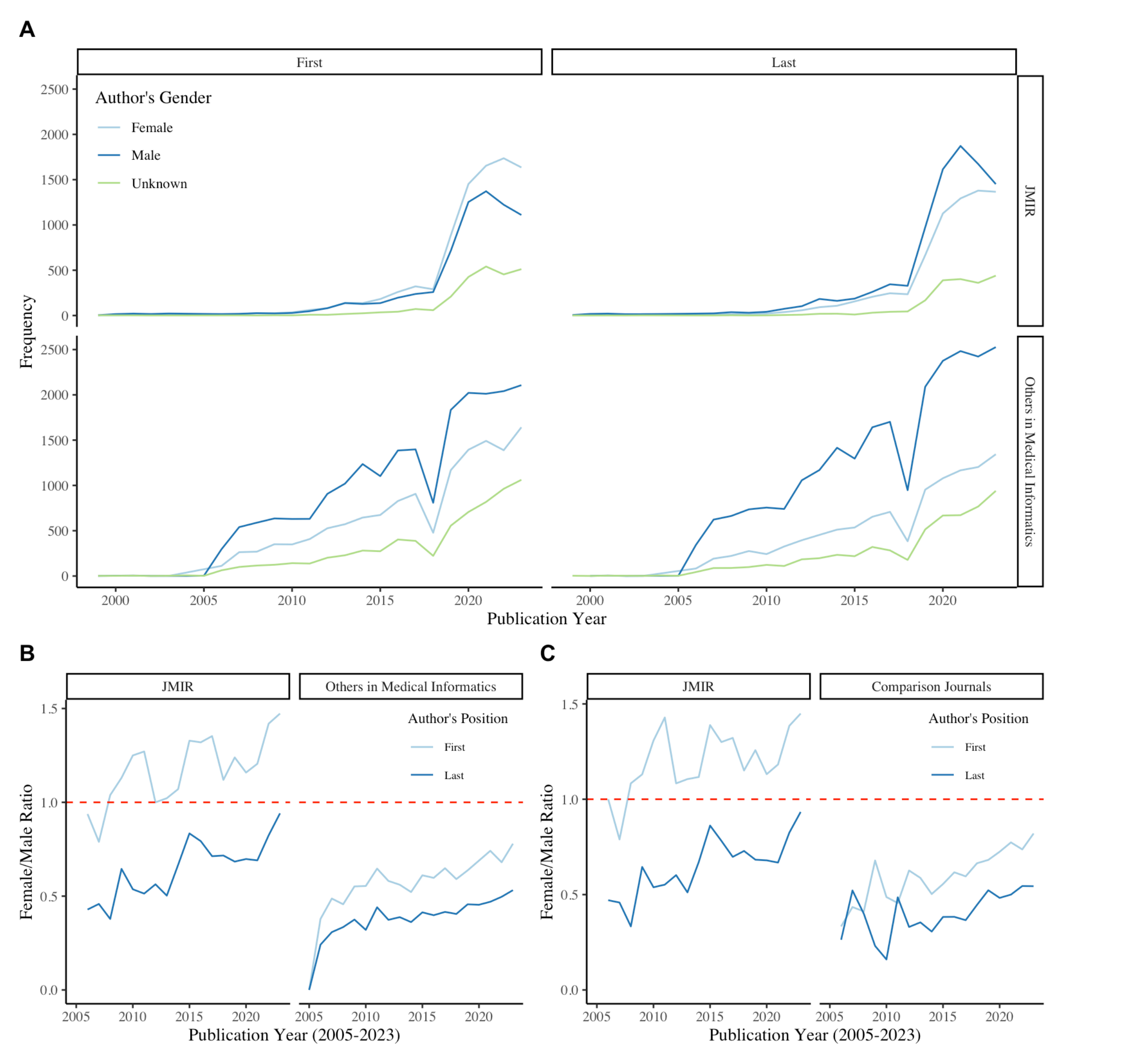

In addition, 7 JMIR Publications journals had more than 50% female first authors and only one had fewer female authors in relevant positions than the pooled analysis of other journals in the field (Figure 4).

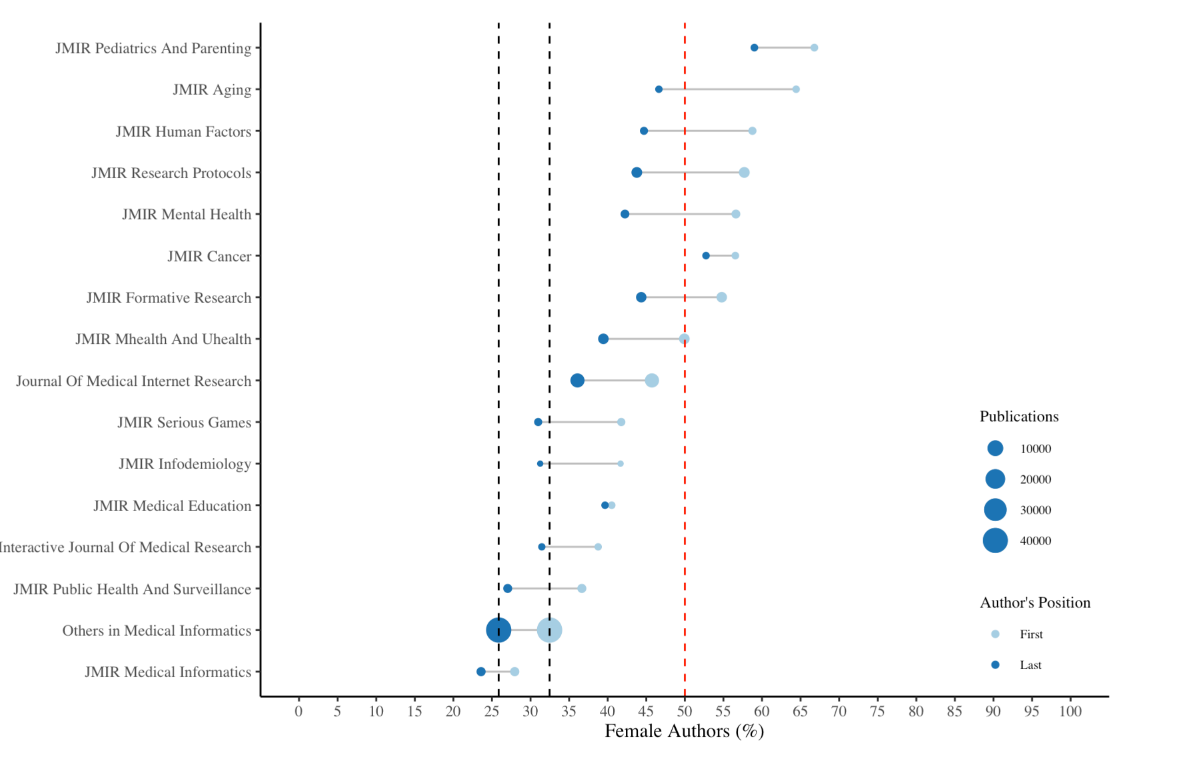

Appendix

1. Appendix: Authorship distribution

Distribution of Authorships

	Within Domain				Within Sample Comparison			
**Characteristic**	**Overall**, N = 59,980[1]	**JMIR**, N = 18,491[1]	**Others in Medical Informatics**, N = 41,489[1]	**p-value**	**Overall**, N = 31,970[1]	**Comparison Journals**, N = 15,985[1]	**JMIR**, N = 15,985[1]	**p-value**
Number of Authors	5.5 ± 4.3	6.7 ± 4.6	5.0 ± 4.0	**<0.001**[2]	6.2 ± 4.3	5.5 ± 3.8	6.8 ± 4.5	**<0.001**[2]
First Author's Gender				**<0.001**[3]				**<0.001**[3]
Female	22,450 / 59,980 (37%)	8,980 / 18,491 (49%)	13,470 / 41,489 (32%)		13,312 / 31,970 (42%)	5,585 / 15,985 (35%)	7,727 / 15,985 (48%)	
Male	28,299 / 59,980 (47%)	7,090 / 18,491 (38%)	21,209 / 41,489 (51%)		13,901 / 31,970 (43%)	7,803 / 15,985 (49%)	6,098 / 15,985 (38%)	
Unknown	9,231 / 59,980 (15%)	2,421 / 18,491 (13%)	6,810 / 41,489 (16%)		4,757 / 31,970 (15%)	2,597 / 15,985 (16%)	2,160 / 15,985 (14%)	
Last Author's Gender				**<0.001**[3]				**<0.001**[3]
Female	17,811 / 59,980 (30%)	7,078 / 18,491 (38%)	10,733 / 41,489 (26%)		10,618 / 31,970 (33%)	4,528 / 15,985 (28%)	6,090 / 15,985 (38%)	
Male	34,463 / 59,980 (57%)	9,458 / 18,491 (51%)	25,005 / 41,489 (60%)		17,487 / 31,970 (55%)	9,296 / 15,985 (58%)	8,191 / 15,985 (51%)	
Unknown	7,706 / 59,980 (13%)	1,955 / 18,491 (11%)	5,751 / 41,489 (14%)		3,865 / 31,970 (12%)	2,161 / 15,985 (14%)	1,704 / 15,985 (11%)	
Gender_concordance				**<0.001**[3]				0.026[3]
Gender Concordance	31,492 / 59,980 (53%)	9,316 / 18,491 (50%)	22,176 / 41,489 (53%)		16,275 / 31,970 (51%)	8,237 / 15,985 (52%)	8,038 / 15,985 (50%)	
Gender Discordance	28,488 / 59,980 (47%)	9,175 / 18,491 (50%)	19,313 / 41,489 (47%)		15,695 / 31,970 (49%)	7,748 / 15,985 (48%)	7,947 / 15,985 (50%)	

[1] Mean and std.-deviation or frequency (%)
[2] Welch Two Sample t-test
[3] Pearson’s Chi-squared test

2. Appendix 2: Matched Regression

Odds Ratio for female authorship in sample comparison

	First Female Authorship					Last Female Authorship				
**Characteristic**	**N**	**Event N**	**OR**[1]	**95% CI**[1]	**p-value**	**N**	**Event N**	**OR**[1]	**95% CI**[1]	**p-value**
**Female in Contrasting Author Position**	31,970	13312	2.37	2.26, 2.49	**<0.001**					
**Gender Concordance**	31,970	13312	0.64	0.61, 0.67	**<0.001**	31,970	10618	1.34	1.27, 1.40	**<0.001**
**JMIR**	31,970	13312	1.74	1.67, 1.82	**<0.001**	31,970	10618	1.56	1.49, 1.63	**<0.001**
**Female in Contrasting Author Position**						31,970	10618	2.37	2.26, 2.49	**<0.001**

[1] OR = Odds Ratio, CI = Confidence Interval

3. Appendix 3: Matching Strategy

Table 3: Sample sizes

	Control	Treated
All (ESS)	41100	18414
All	41100	18414
Matched (ESS)	15985	15985
Matched	15985	15985
Unmatched	25115	2429
Discarded	0	0

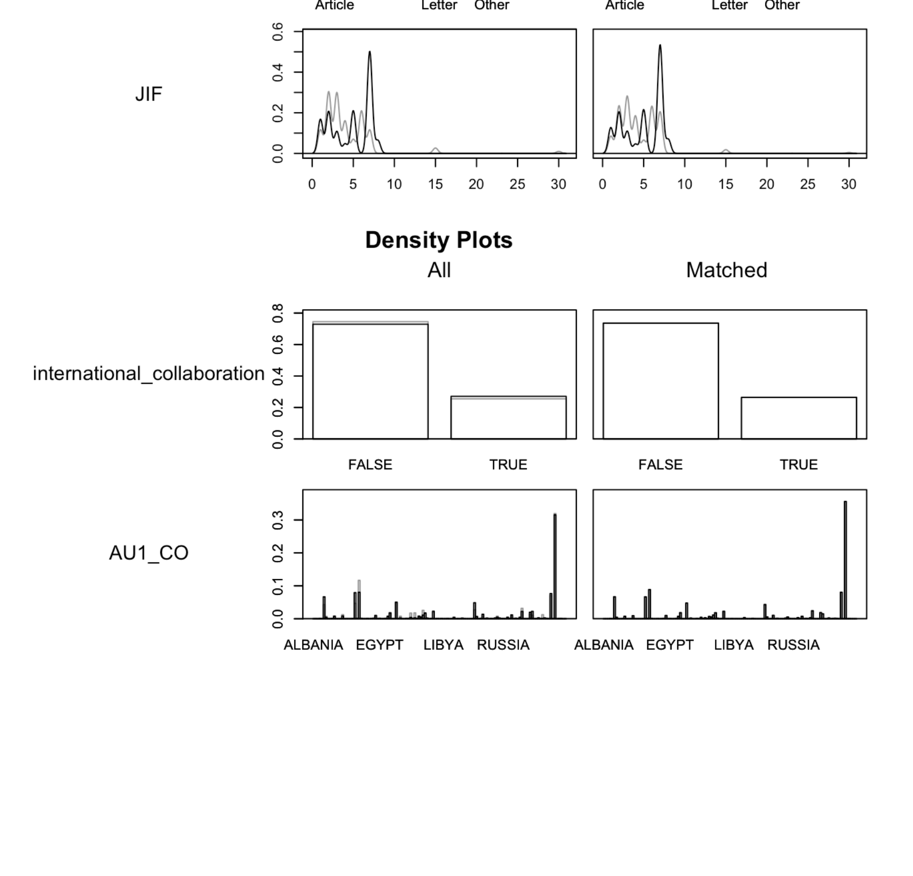

Supplement: Multimedia Appendix 2 [file jmir_v26i1e58950_app2.pdf]
