# Supplementary material for: Twenty-Five Years of Progress—Lessons Learned From JMIR Publications to Address Gender Parity in Digital Health Authorships: Bibliometric Analysis
Source: J Med Internet Res. 2024 Aug 9;26:e58950. doi: 10.2196/58950 (PMC11344179; doi:10.2196/58950)
Supplement: Multimedia Appendix 3 [file jmir_v26i1e58950_app3.docx]

| **Characteristic** | **Distribution of Authorship within Domain** | | | | **Distribution of Authorship within Sample Comparison** | | | |
| --- | --- | --- | --- | --- | --- | --- | --- | --- |
|  | **Overall**, N = 59,980*^a^* | **JMIR**, N = 18,491 *^a^* | **Others in Medical Informatics**, N = 41,489 *^a^* | **p-value** | **Overall**, N = 37,643 *^a^* | **Comparison Journals**, N = 22,289 *^a^* | **JMIR**, N = 15,354 *^a^* | **p-value** |
| Number of Authors | 5.5 ± 4.3 | 6.7 ± 4.6 | 5.0 ± 4.0 | **<0.001***^2^* | 6.0 ± 4.4 | 5.5 ± 4.3 | 6.8 ± 4.5 | **<0.001***^b^* |
| First Author's Gender |  |  |  | **<0.001***^3^* |  |  |  | **<0.001***^c^* |
| Female | 22,450 / 59,980 (37%) | 8,980 / 18,491 (49%) | 13,470 / 41,489 (32%) |  | 14,826 / 37,643 (39%) | 7,204 / 22,289 (32%) | 7,622 / 15,354 (50%) |  |
| Male | 28,299 / 59,980 (47%) | 7,090 / 18,491 (38%) | 21,209 / 41,489 (51%) |  | 16,378 / 37,643 (44%) | 10,628 / 22,289 (48%) | 5,750 / 15,354 (37%) |  |
| Unknown | 9,231 / 59,980 (15%) | 2,421 / 18,491 (13%) | 6,810 / 41,489 (16%) |  | 6,439 / 37,643 (17%) | 4,457 / 22,289 (20%) | 1,982 / 15,354 (13%) |  |
| Last Author's Gender |  |  |  | **<0.001***^3^* |  |  |  | **<0.001***^c^* |
| Female | 17,811 / 59,980 (30%) | 7,078 / 18,491 (38%) | 10,733 / 41,489 (26%) |  | 11,913 / 37,643 (32%) | 5,884 / 22,289 (26%) | 6,029 / 15,354 (39%) |  |
| Male | 34,463 / 59,980 (57%) | 9,458 / 18,491 (51%) | 25,005 / 41,489 (60%) |  | 20,284 / 37,643 (54%) | 12,533 / 22,289 (56%) | 7,751 / 15,354 (50%) |  |
| Unknown | 7,706 / 59,980 (13%) | 1,955 / 18,491 (11%) | 5,751 / 41,489 (14%) |  | 5,446 / 37,643 (14%) | 3,872 / 22,289 (17%) | 1,574 / 15,354 (10%) |  |
| Gender_concordance |  |  |  | **<0.001***^3^* |  |  |  | 0.681*^c^* |
| Gender Concordance | 31,492 / 59,980 (53%) | 9,316 / 18,491 (50%) | 22,176 / 41,489 (53%) |  | 19,046 / 37,643 (51%) | 11,297 / 22,289 (51%) | 7,749 / 15,354 (50%) |  |
| Gender Discordance | 28,488 / 59,980 (47%) | 9,175 / 18,491 (50%) | 19,313 / 41,489 (47%) |  | 18,597 / 37,643 (49%) | 10,992 / 22,289 (49%) | 7,605 / 15,354 (50%) |  |
| *^a^* Mean and std.-deviation or frequency (%) | | | | | | | | |
| *^b^* Welch Two Sample t-test | | | | | | | | |
| *^c^* Pearson’s Chi-squared test | | | | | | | | |
