# Supplementary material for: Twenty-Five Years of Progress—Lessons Learned From JMIR Publications to Address Gender Parity in Digital Health Authorships: Bibliometric Analysis
Source: J Med Internet Res. 2024 Aug 9;26:e58950. doi: 10.2196/58950 (PMC11344179; doi:10.2196/58950)
Supplement: Multimedia Appendix 4 [file jmir_v26i1e58950_app4.docx]

| **Characteristic**,  N = 37,643 | **First Female Authorship**,  N = 14,826 | | | **Last Female Authorship**,  N = 11,913 | | |
| --- | --- | --- | --- | --- | --- | --- |
|  | **OR***^a^* | **95% CI***^a^* | **p-value** | **OR***^a^* | **95% CI***^a^* | **p-value** |
| Female in Contrasting Author Position | 2.46 | 2.35, 2.57 | <0.001 | 2.46 | 2.35, 2.57 | <0.001 |
| Gender Concordance | 0.63 | 0.60, 0.65 | <0.001 | 1.24 | 1.19, 1.29 | <0.001 |
| JMIR | 2.06 | 1.98, 2.15 | <0.001 | 1.80 | 1.73, 1.88 | <0.001 |
| ^a^ OR = Odds Ratio, CI = Confidence Interval | | | | | | |
